# Supplementary figures and images for: Differences in phenotype between long-lived memory B cells against Plasmodium falciparum merozoite antigens and variant surface antigens
Source: PLoS Pathog. 2024 Oct 28;20(10):e1012661. doi: 10.1371/journal.ppat.1012661 (PMC11542837; doi:10.1371/journal.ppat.1012661)

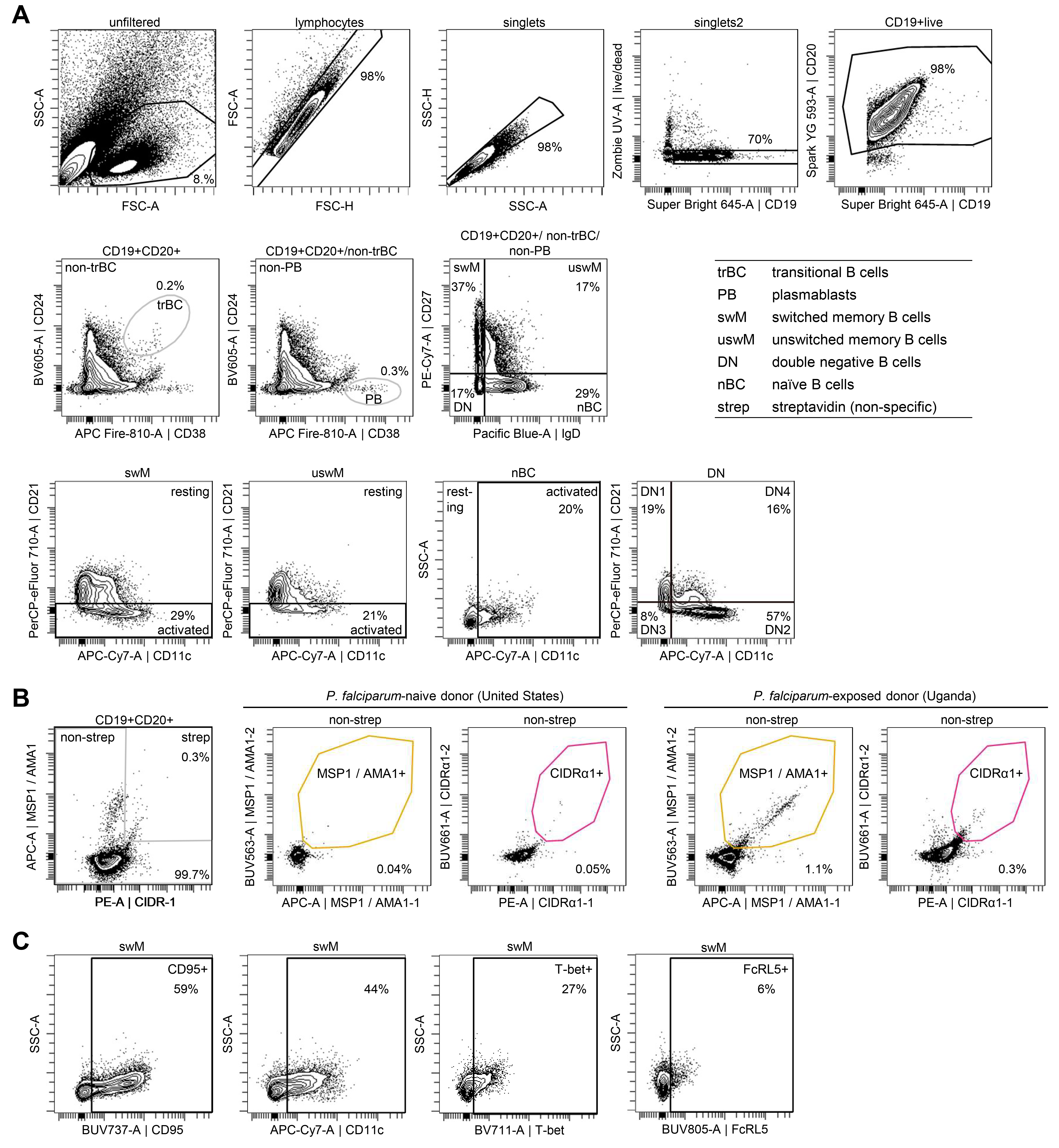

Supplement: S1 Fig — A) The gating scheme used to identify resting and activated B cell populations, as well as subpopulations of double negative cells. The table lists all abbreviations of B cell populations and their definitions. B) The gating strategy used to identify antigen-specific B cells. Left: cells that bound to both CIDRα1 and MSP1/AMA1 tetramers (denoted as “strep”) were considered non-specific binders. Only “non-strep” cells were used to gate on antigen-specific B cells. Middle: background of antigen-specific B cells in a P. falciparum-naïve US donor. Right: detection of antigen-specific B cells in a P. falciparum-exposed Ugandan donor. C) The gating strategy used to identify CD95+, CD11c+, T-bet+, and FcRL5+ B cells. (TIF) [file ppat.1012661.s001.tif]

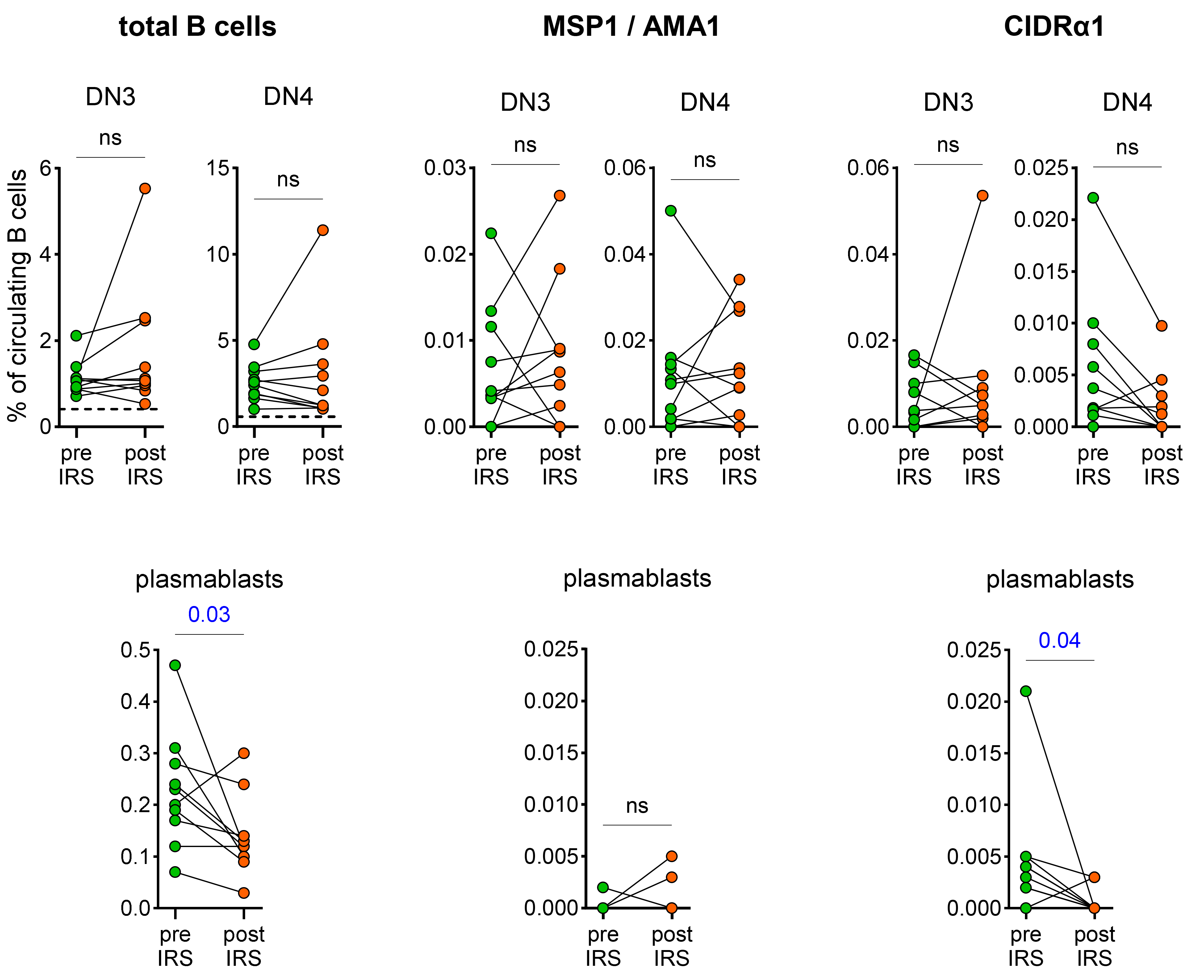

Supplement: S2 Fig — The percentages are shown for total B cells (left), MSP1/AMA1-specific B cells (middle), and CIDRα1-specific B cells (right). Differences between groups were evaluated using a Wilcoxon matched-pairs signed-rank test. (TIF) [file ppat.1012661.s002.tif]

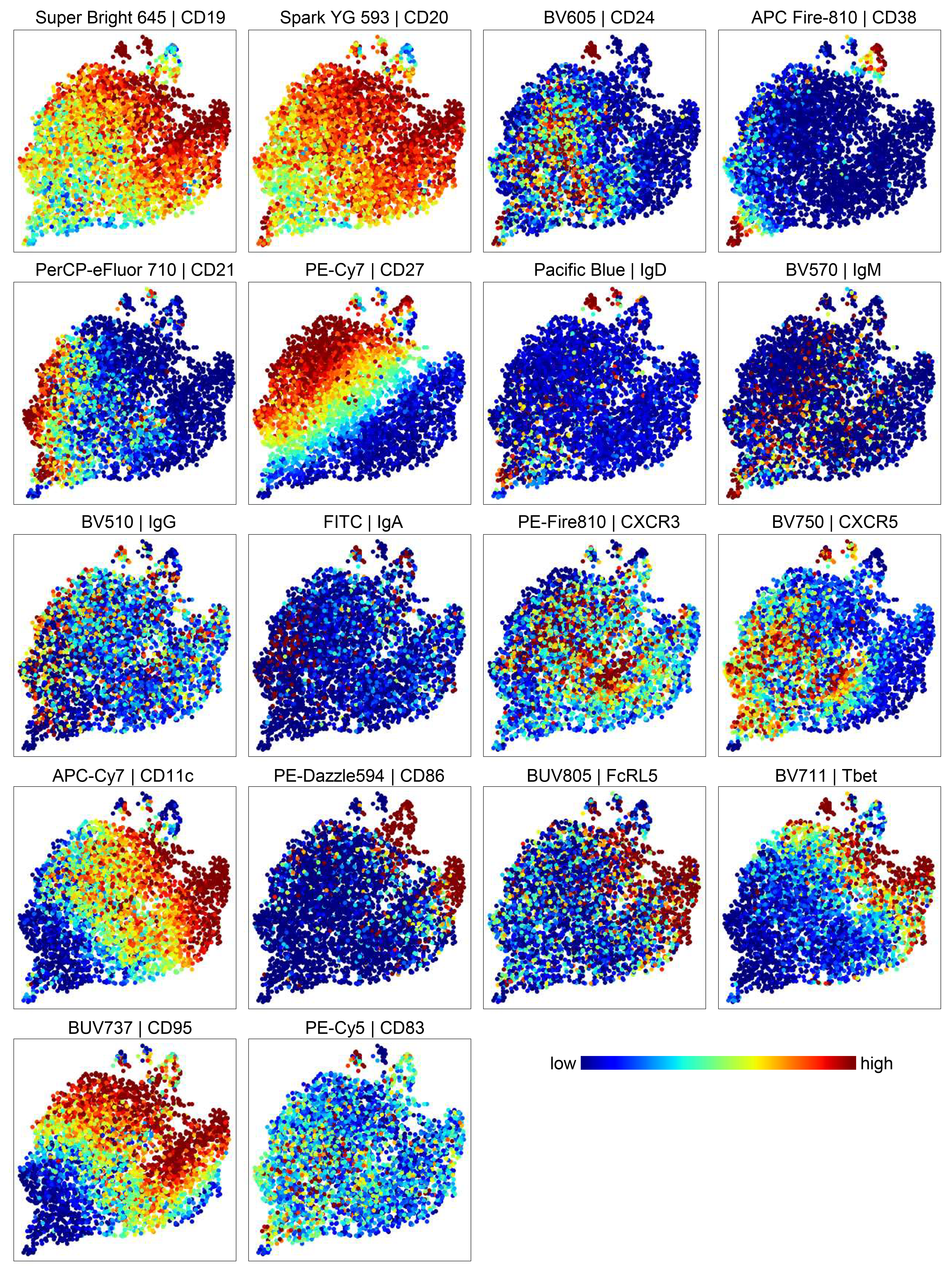

Supplement: S3 Fig — (TIF) [file ppat.1012661.s003.tif]

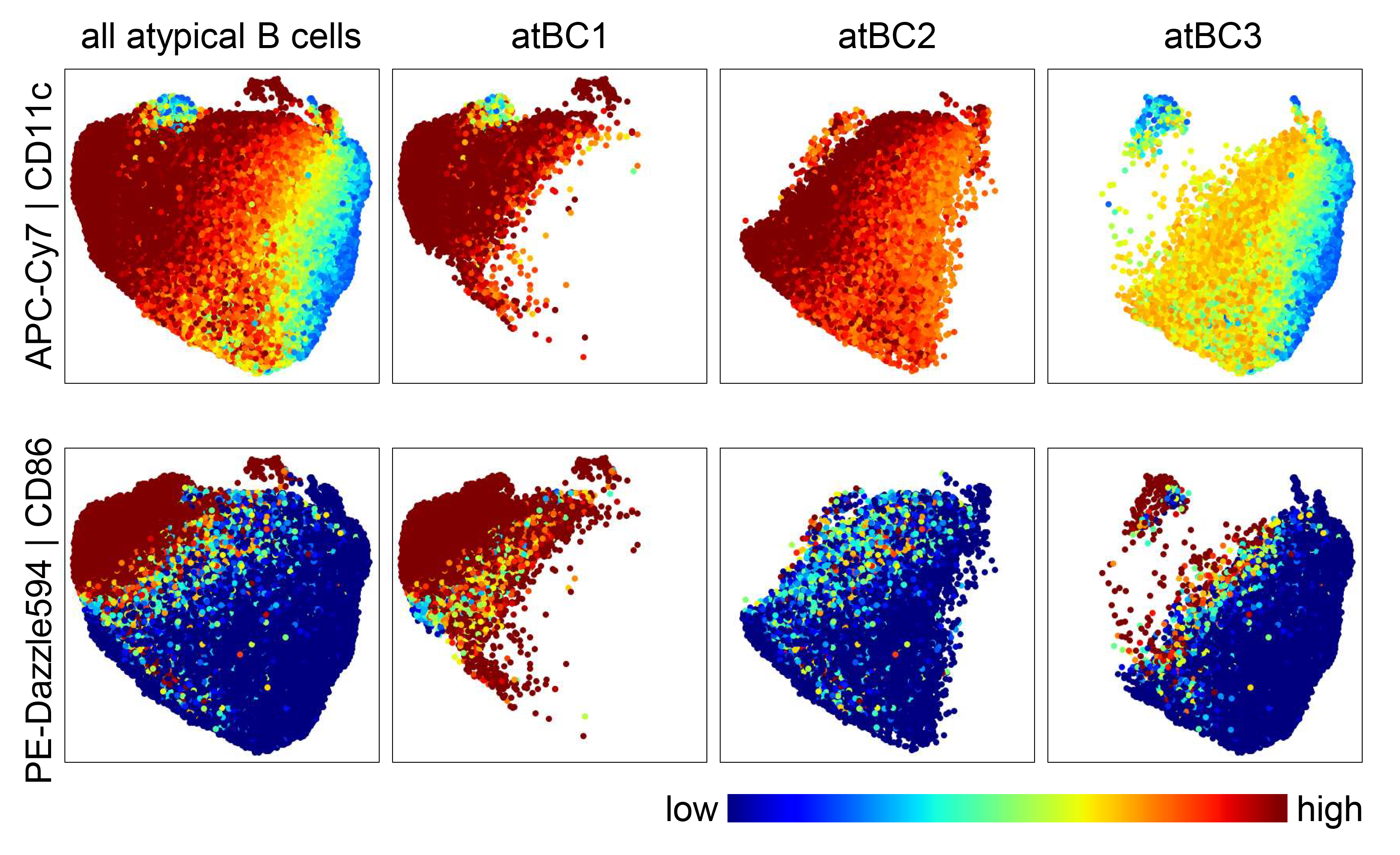

Supplement: S4 Fig — (TIF) [file ppat.1012661.s004.tif]

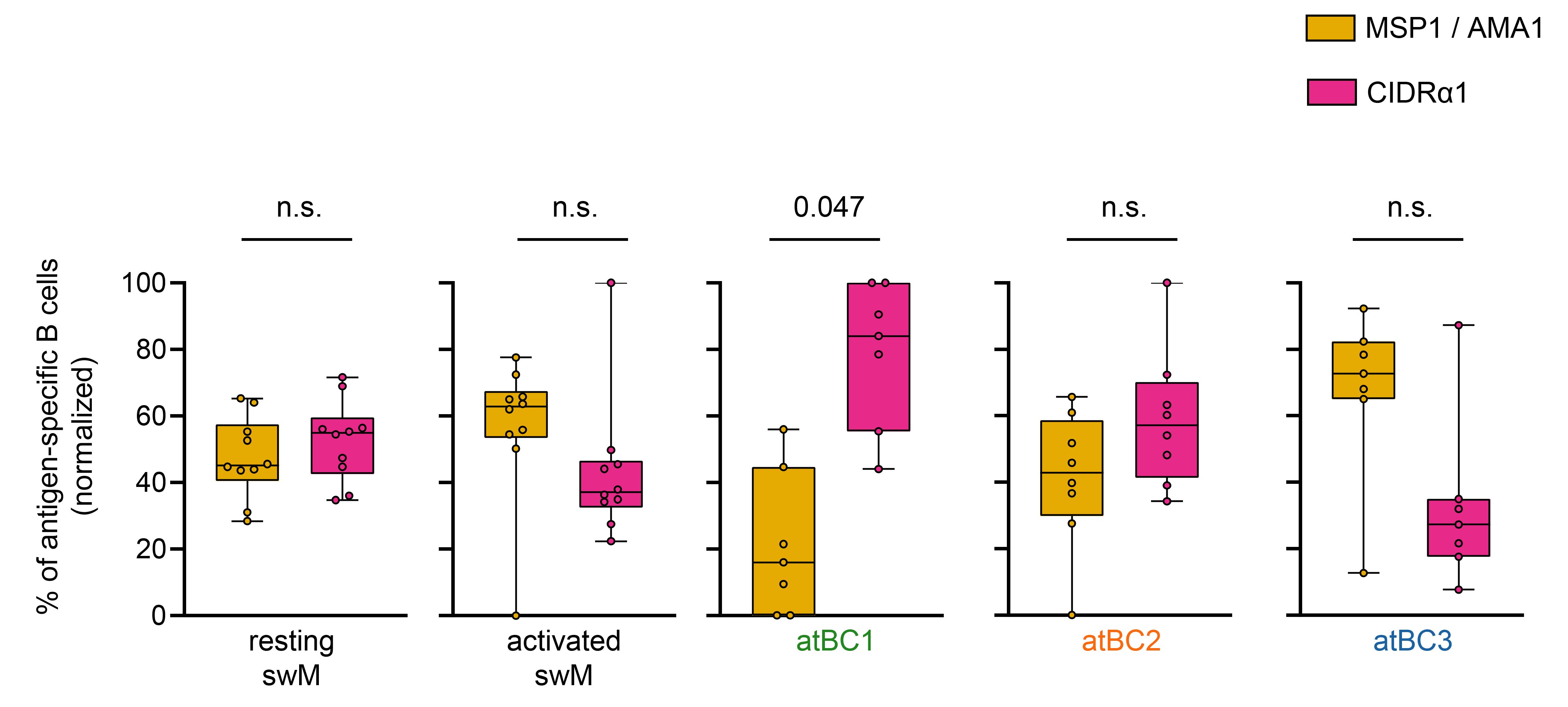

Supplement: S5 Fig — Values were calculated for individual donors. For the three atypical B cell subsets, donors were only included if the total size of the subset allowed for the detection of at least one MSP1/AMA1-specific B cell and one CIDRα1-specific B cell. For example, if the total percentage of MSP1/AMA1-specific B cells is 1.5%, a B cell population needs to contain at least 1 / 1.5 × 100 = 67 cells to be included in this analysis. Because atypical B cell subsets in samples obtained post-IRS were too small to perform this analysis, only pre-IRS data is shown. swM, switched memory B cell; atBCi, atypical B cell subset i. n.s., not significant. (TIF) [file ppat.1012661.s005.tif]
