# Supplementary material for: Differences in phenotype between long-lived memory B cells against Plasmodium falciparum merozoite antigens and variant surface antigens
Source: PLoS Pathog. 2024 Oct 28;20(10):e1012661. doi: 10.1371/journal.ppat.1012661 (PMC11542837; doi:10.1371/journal.ppat.1012661)
Supplement: Attachment — Submitted filename: 147694_1_rebuttal_3117957_s3ft3y.docx [file ppat.1012661.s009.docx]

**Manuscript number:** RC-2024-02536

**Corresponding author(s):** Evelien Bunnik

## 1. General Statements [optional]

## 2. Point-by-point description of the revisions

We would like to thank the reviewers for their overall positive assessment of our manuscript. We have used their constructive feedback to substantially improve our manuscript as described below. Changes in the main manuscript are indicated in red.

**Reviewer #1**

**Evidence, reproducibility and clarity**

This study by Reyes at al is a well conducted analysis of memory B cell dynamics of Plasmodium falciparum (Pf) -specific B cell populations over the course of reducing Pf prevalence in ten Ugandan adults. The data is presented well and the authors provide compelling evidence that 1. There is an overall loss of Ag specific B cells with reduction in exposure and 2. Different antigens (MSP1/AMA-1 vs CIDRa-1) generate different flavors of long lived responses. However, additional clarity to the reader should be provided on certain topics (listed below).

**Major comments:**
1. While the premise of the study (reduced Pf transmission due to the use of indoor residual spraying (IRS)) is an important one, I think the authors must take into consideration that 9/10 subjects had at least one Pf positive episode between Time Points 1 and 2 (Figure 1). Also, it looks from Fig 1 that some samples were collected at a time of Pf positive test (green squares), while in Table S1 none of the subjects have a positive parasite status at TP1.

We recognize that most individuals had detectable parasitemia before and after time point (TP) 1. In our manuscript, we therefore do not report the time between TP1 and TP2, because we agree that the length of this time interval is not relevant in our study methodology. We only mention the time between the last known *P. falciparum* infection and collection of blood at the second time point. We use the sample collected at TP1 only as a representative sample obtained during a time with high *P. falciparum* exposure and do not make any claims based on the time between TP1 and TP2. The occurrence of infections after sample collection at TP1 confirms that parasite transmission was still high at this time. We have added a schematic of the relative levels of parasite transmission to Figure 1 to emphasize this.

With respect to infection status, none of the donors were blood smear positive at TP1. However, as mentioned in Table S1, parasites were detected in three individuals using the more sensitive LAMP assay. These three individuals are therefore marked as parasite positive in Figure 1. Table S1 has been modified to highlight the parasite status of these three individuals.

| **Donor ID** | **Parasite status at TP1** |
| --- | --- |
| 3151 | Negative blood smear / LAMP negative |
| 3188 | Negative blood smear / LAMP positive |
| 3212 | Negative blood smear / LAMP negative |
| 3244 | Negative blood smear / LAMP negative |
| 3261 | Negative blood smear / LAMP negative |
| 3283 | Negative blood smear / LAMP negative |
| 3338 | Negative blood smear / LAMP negative |
| 3223 | Negative blood smear / LAMP negative |
| 3324 | Negative blood smear / LAMP positive |
| 3341 | Negative blood smear / LAMP positive |

2. Figure S1A: What is trBC? Figure S1B: What is Strep? Are the strep positive cells also CIDR-1 positive and were they gated out? Why is APC used for MZ-1 and one of the MSP1-AMA-1 tetramers? Do these stainings come from multiple panels?

All abbreviations of B cell populations were defined in the figure legend (for example, trBC stands for transitional B cells). To facilitate the interpretation of Figure S1, we have now included the definitions of these abbreviations in the figure.

Strep stands for streptavidin, which has now also been clarified in the figure. In our gating strategy, we used the term “strep” to denote cells that bound to both CIDRa1 and MSP1/AMA1 tetramers, which we interpreted as non-specific binding to streptavidin or other components of the antigen tetramers. Only the “non-strep” cells were used to gate on antigen-specific cells. We have added this clarification to the figure legend.

In panel B, we accidentally used the term MZ (for merozoite) to describe tetramers of the merozoite antigens MSP1 / AMA1. These labels are interchangeable, but to avoid confusion, MZ-1 has been changed to MSP1 / AMA1.

3. Figure 3A: how many cells does the umap plot represent? Were there a total of 3555 Ag specific B cells that were non-naive (Figure 3E)?

It is correct that there were a total of 3,555 antigen-specific B cells used for the clustering shown in panel A. This information has been added to Figure 3A.

4. Could the authors comment on why in Figure 3, Ig isotype expression was not considered for clustering? This would allow for characterization of DN sub populations/ clusters in addition to the CD21-CD27- ABCs? It looks like IgD expression was low across the clusters (Figure 3D). Was this the case for the cells considered in this analysis, or was it excluded? If it was truly low expressed, how were the assessments in Figure 2 made?

From prior experience, we know that Ig isotype information tends to dominate in the clustering, which would result in major clusters based on IgM, IgD, IgG, and IgA expression, not on expression of other markers. This is illustrated in the example below. The UMAP on the left shows clusters in green and red that consist of IgG^+^ and IgA^+^ B cells, respectively. The UMAP on the right shows that switched memory (swM) B cells and DN B cells are found in both IgG and IgA clusters. Because we were mainly interested in identifying different subsets of B cells, irrespective of Ig isotype, we did not include Ig isotype in the clustering. We have clarified in the manuscript that Ig isotypes were excluded from the analysis to prevent these from dominating the clustering:

“Unsupervised clustering was then performed based on expression of all markers, except for Ig isotypes to prevent these from dominating the clustering.”

from: Reyes et al., Plos ONE, 2021, https://doi.org/10.1371/journal.pone.0261656

IgD expression among cell clusters shown in Figure 3 was low because only non-naïve B cells were included in the analyis. The majority of non-naïve cells are class-switched memory B cells and DN B cells, which by definition do not express IgD (see gating strategy in Figure S1A). Figure 2 shows all B cell populations, including naïve B cells and non-naïve B cell populations (unswitched memory, switched memory, and DN), that were gated based on IgD and CD27 expression.

5. Are there differences in these designations / phenotypes of DN populations of atBCs vs CD21-CD27- atBCs?

In the malaria field, atypical B cells are typically defined as CD21^-^CD27^-^. The definition of DN2 B cells comes from the autoimmunity field and is stricter: IgD^-^CD27^-^CD21^-^CD11c^+^ B cells. In our manuscript, we define atypical B cells in a stricter way than typically done in the malaria field, following published guidelines for the identification of B cell subsets (https://doi.org/10.3389/fimmu.2019.02458). Using these guidelines, atypical B cells and DN2 B cells are phenotypically identical. We have added a reference to these published guidelines in the Results section:

“Following published guidelines for the identification of B cell populations (21), total CD19^+^ B cells were divided into naïve B cells (IgD^+^CD27^-^), unswitched memory B cells (IgD^+^CD27^+^), switched memory B cells (IgD^-^CD27^+^), and double negative B cells (IgD^-^CD27^-^).”

6. Lines 258-259: In considering only switched MBCs, what clusters from Figure 3a were included? There seem to be 2588 sw MBCs (Table S3, Figure 4). Do the remaining cells (967 cells) come from clusters 2, 5 and 6 (and excludes the atBC clusters)

This analysis did not use the clusters presented in Figure 3, but instead used switched memory B cells gated as shown in Figure S1A. The reason for this is that the clusters in Figure 3 were generated using antigen-specific B cells and cannot be reproduced using non-antigen-specific B cells. Thus, it is not possible to separate all other B cells into the same six clusters. The only way to compare expression of certain markers between antigen-specific and non-antigen specific switched memory B cells is to gate on these populations manually. We have now tried to clarify this in the manuscript as follows:

“we determined the percentages of CD95^+^ cells and CD11c^+^ cells among antigen-specific switched memory B cells and the total population of switched memory B cells (gated manually as shown in Figure S1A).”

**Minor comments:**
1. Line 178- 179: Was there a specific measure of rate of decline made for these cells?

We did not calculate a rate of decline of antigen-specific B cells for several reasons: 1) the time between TP1 and TP2 is not the same for all people in the study, 2) the time between last exposure and TP2 is not the same for all people, and 3) the rate of decline is most likely not linear and cannot accurately be estimated with only two data points. We have changed the wording of this sentence such that we do not use the word “rate”:

“we did not observe a difference in the percentage of B cells with specificity for merozoite antigens or variant surface antigens that were lost.”

In addition, we included the percentage of reduction in size in the paragraph before this section:

“we observed that both populations decreased in size by about 50%, although these differences were not statistically significant.”

**Significance**

General assessment:
Strengths: The authors provide evidence that the dynamics of antigen specific cells in humans can vary with exposure and with the nature of the antigen. They have nicely discussed the potential causes for these differences (Discussion), although they should include the findings of Ambegaonkar et al that ABCs in malaria may be restricted to responding specifically to membrane bound antigens (PMCID: PMC7380957)

As suggested by the reviewer, we have added a paragraph to the Discussion section to discuss the results reported by Ambegaonkar et al. and how the difference between soluble vs. membrane-bound antigens may have an effect on how these antigens are perceived by B cells:

The difference between soluble and membrane-bound antigens may also have a direct effect on how these antigens are perceived by B cells. Atypical B cells have been shown to be restricted to recognition of membrane-bound antigens (41). The interaction of a B cell with membrane-associated antigen allows the formation of an immunological synapse. Inhibitory receptors expressed by atypical B cells are excluded from this synapse, resulting in B cell receptor signaling and differentiation towards antibody-secreting cells (41). This could explain why atypical B cell subset 1 that expresses the highest levels of the inhibitory receptor FcRL5 is enriched for recognition of the CIDRα1 domain of membrane-bound protein PfEMP1. It should however be noted that soluble antigen can also be presented effectively in membrane-context by conventional dendritic cells, follicular dendritic cells, and subcapsular macrophages in secondary lymphoid organs, especially when it is part of an immune complex (reviewed in (42)). This would provide a route for atypical B cells to also respond to soluble merozoite antigens, such as MSP1 and AMA1.

Limitations:
1. Outlined above, and as the authors also mention, a small sample size and homogenous population.
2. The evidence for reduced transmission is not clear, and the negative parasite tests for donors shown in Table S1 do not match with Figure 1 data.
3. Lack of IgD expression across clusters (Figure 3D- the authors will need to clarify this point) would require re-analysis of Figure 2 data

1. We have provided clarification in response to the points raised by the reviewer.

2. We believe there is clear evidence for reduced transmission, from a median of almost 2 infections per person per year prior to the implementation of IRS to a median parasite-free period of 1.7 years prior to sample collection at TP2. To further emphasize this, we have summarized the number of *P. falciparum* infections among the ten individuals included in this study (now included in Table S3):

| year | *Pf* infections | comment |
| --- | --- | --- |
| 2012 | 20 |  |
| 2013 | 19 | TP1 |
| 2014 | 20 | TP1 |
| 2015 | 8 | Start IRS |
| 2016 | 0 | TP2 |

This reduced parasite exposure is reflected in a decrease in immune activation as presented in Figure 2. We have clarified that the data in Table S1 did indeed match those shown in Figure 1.

3. We have clarified that IgD expression is low in the clusters presented in Figure 3 because naïve B cells were excluded from this analysis.

Advances: This study highlights the importance of studying antigen specific B cells in humans in the context of natural infection and the use of high-parameter tools such as spectral flow cytometry in assessing a large quantity of data from limited clinical samples. These data are important to inform better vaccine design. Studies in inbred animals can be quite limited or different from human B cell responses.

Audience: This study will be of interest to malariologists and B cell immunologists. Atypical B cells are relevant to many infectious diseases and auto immunity, while the dynamics of memory B cells in malaria all be relevant to those interested in vaccine design against blood stage antigens.

**Reviewer #2**

**Evidence, reproducibility and clarity**

Summary:
In this study, the authors compared long-lived total and antigen (ag)-specific B-cell levels in a cohort of 10 Ugandan malaria patient samples that were collected before and after local reduction of P. falciparum transmission (pre/post-IRS). The focus is on the novel comparison of the two most common malaria antigens: merozoite antigens (MSP1/AMA1) and variant surface antigens (CIDRα1). Using high-parameter spectral flow cytometry, they also characterized the phenotype of the different populations of cells. Their main findings include 1) a decrease in activated but maintenance of resting ag-specific B-cells in the post-IRS sample and 2) CD95 and CD11c, as the only differentially expressed markers between MSP1/AMA1-specific and CIDRα1-specific long-lived memory B cells. Their further phenotypic characterization suggests functional consequences with MSP1/AMA1-specific B-cells being poised for rapid antibody-secreting cell differentiation while CIDRα1-specific B cells were enriched among a subset of atypical B cells that seem poised for antigen presentation (CD86+CD11chi/ AtBC1).
Their findings consolidate and further expand our knowledge of long-lived B-cell levels during P. falciparum malaria and report/compare (for the first time to my knowledge) a differential selection of long-lived B-cell levels between these 2 antigen specificities. Overall, the manuscript is straightforward and well-written and the authors did a good job explaining their methodology, findings, and interpretations.
I believe the major gap missing in this study is the reconciliation of long-lived antigen-specific B-cell levels with the serum antigen-specific antibody levels of these patients against the same 2 antigens (MSP1/AMA1 and CIDRα1) in the experiments and the discussion. The antibody data would strengthen their main argument and is the main missing piece for characterizing more completely the long-lived antigen-specific humoral responses. Below are my suggestions that would help improve the manuscript:

**Major comments:**
1. Serum Anti-Pf antibodies: Do the authors have access to the serum/plasma of these patients? It would be important to correlate the total and ag-specific B-cell populations with levels of serum IgG antibodies against those specific Pf antigens (MSP1/AMA1 and CIDRα1) and total IgG levels to strengthen their point about long-lived humoral responses.

To our understanding, the rationale for such an analysis would be that if IgG levels correlated with the size of a certain B cell population, it would suggest that this B cell population is implicated in the production of IgG against a particular antigen. While a correlation between the percentage of memory B cells and IgG titers has been observed for antigens from several viruses and bacteria (1-4), other studies have reported the absence of such a correlation (4-7). Similarly, for *P. falciparum* antigens, a moderate correlation between memory B cell abundance and IgG titers has been observed for some merozoite antigens, but not for others (8, 9). The lack of a correlation between the magnitude of the memory B cell and the antibody response fits with the prevailing model that memory B cells and plasma cells are two independently controlled arms of the humoral immune system (10, 11). Given the lack of strong evidence that the levels of IgG titers and memory B cells are interconnected, we do not think this analysis will be informative.

An alternative analysis would be to study the contribution of B cell subsets to the production of IgG after re-exposure, similar to a recent study that identified T-bet^+^ memory B cells as the main contributors to antibody responses following influenza virus vaccination (12). Unfortunately, we are unable to perform this analysis in this study population, because only four of the individuals included in this study (spanning calendar years 2012 – 2016) were recruited into a follow up cohort (calendar years 2017 – 2019), and none of these four people were infected during this later time frame.

We have however added this future direction to the Discussion section:

To determine the contribution of different memory B cell subsets to the recall response against *P. falciparum*, it would be interesting to analyze IgG responses upon re-infection. However, none of the individuals included in this study experienced a recorded *P. falciparum* infection post-IRS, preventing us from performing such an analysis.

References

1. Crotty et al., J Immunol (2003), https://doi.org/10.4049/jimmunol.171.10.4969
2. Quinn et al., J Infect Dis (2004), https://doi.org/10.1086/423937
3. Cohen et al., Cell Rep Med (2021), <https://doi.org/10.1016/j.xcrm.2021.100354>
4. Amanna et al., New England J Med (2007), https://doi.org/10.1056/nejmoa066092
5. Leyendeckers et al., Eur J Immunol (1999), https://doi.org/10.1002/(sici)1521-4141(199904)29:04%3C1406::aid-immu1406%3E3.0.co;2-p
6. Nanan et al., Vaccine (2001), https://doi.org/10.1016/s0264-410x(01)00328-0
7. Goel et al., Science Immunol (2021), https://doi.org/10.1126/sciimmunol.abi6950
8. Rivera-Correa et al., eLife (2019), https://doi.org/10.7554/elife.48309
9. Jahnmatz et al., Front Immunol (2021), https://doi.org/10.3389/fimmu.2020.619398
10. Weisel et al., Immunity (2016), https://doi.org/10.1016/j.immuni.2015.12.004
11. Shinnakasu et al., Nat Immunol (2016), https://doi.org/10.1038/ni.3460
12. Nellore et al., Immunity (2023), https://doi.org/10.1016/j.immuni.2023.03.001

2. Correlation between populations and initial parasite load: Are the levels between any of the populations at any time point correlated significantly in any way? If the statistical power/N allows it, please perform a correlation array between all populations using all samples both total and ag-specific and initial parasite load.

We agree that this analysis could be very interesting. However, in most recorded infection cases, parasitemia was submicroscopic and parasite load was not reported. Information about parasite density in the blood prior to TP1 is available for only half of the individuals in this study. In these people, the last known parasite density was recorded between three months to two years prior to TP1. Given the small number of individuals for whom these data are available and the large variation in time between parasitemia and sampling, we do not have sufficient data to perform this analysis.

3. Figure 2: Why were total and ag-specific plasmablasts/plasma cells not included in this figure? Please include to compare levels in these two time points.

We did not include the levels of total and antigen-specific plasmablasts (PBs) in Figure 2 because the percentages of PBs are relatively low, and very few antigen-specific PBs were detected. We have now included the levels of total PBs in Figure 2A and the percentages of antigen-specific PBs in Supplementary Figure 2. The percentage of PBs among total B cells decreased by about 50% between TP1 and TP2, in line with a decrease in immune activation.

4. Healthy baseline: The study is missing "healthy" controls as a reference. I presume this is because each patient is its uninfected control in the post-IRS sample. In methods, they mentioned they used two naïve-USA B-cells as technical controls. It would be important to include and maybe expand (to match age and gender)on that specific data from those controls as supplementary figures to support their findings:
1. Show negative Tetramer staining for these samples (to understand the background).
2. Levels of all the USA controls total B cell populations and compared to the pre/post-IRS samples to understand "baseline" or "non-endemic" control levels.

1. We have included flow cytometry plots of tetramer staining for the non-*P. falciparum* exposed donors (pooled B cells from two US donors) to show the level of background for these probes. These plots are shown in Figure S1B.

2. We have used data from *P. falciparum*-naive US donors (n = 7) that we generated for a prior study to show the average level of total B cell populations in Figure 2, and the percentage of switched memory B cells that express CD95, CD11c, T-bet, and FcRL5 in Figure 4.

**Minor comments:**
1. In the gating strategy (S1), please include the percentage of each population of that representative example.

We have added the percentages for all gated populations to Figure S1.

2. For Figure 2, since not every panel has the same N, please include the N for each panel in the figure or a supplementary table.

All panels in Figure 2 show data for all 10 individuals. However, since some data points are overlapping, it may appear that some panels show data from fewer individuals. Specifically, no antigen-specific DN1 cells were detected pre- and post-IRS for four individuals. These data points therefore overlap and are not visible. To avoid confusion, we had mentioned this in the legend to Figure 2 (see text in orange). We have tried to further clarify this by emphasizing in the figure legend that data from all 10 individuals are shown (see text in red):

“**Figure 2: Abundance of total and antigen-specific B cell subsets in the circulation during high parasite transmission and in the absence of P. falciparum exposure.** The percentage of B cell subsets among circulating B cells is shown for total B cells (A), MSP1/AMA1-specific B cells (B), and CIDRα1-specific B cells (C). For MSP1/AMA1-specific B cells and CIDRα1-specific B cells, the total percentage among all circulating B cells is also shown (right most graphs in each panel). All panels show data for all 10 individuals. In panels B and C, no antigen-specific DN1 cells were detected pre- and post-IRS for four individuals. These data points therefore overlap and are not clearly visible. Differences between groups were evaluated using a Wilcoxon matched-pairs signed-rank test. P values < 0.05 are shown in blue. Ns, not significant.”

3. Please mention the history of past and chronic co-infections of these 10 patients. Particularly if they had any other active or recent infection when the sample was taken.

Four individuals had active or recent infections in the three months prior to sample collection, with upper respiratory tract infections being the most common. This information has been included in Table S3, with a reference to these data in the Methods section. We have also included a link to ClinEpiDB where additional information about the cohort participants, including medical history, can be found.

4. Discussion: further discussion with relevant literature on the following points is needed to consolidate cellular and antibody studies:
a. Whether the presence of long-lived ag-specific B-cell responses correlates with sustained levels of IgG against Pf antigens.
b. The different types of antibodies (protective/pathogenic) that these different B-cell populations have been reported to produce during malaria.

a. We have added the following paragraph to the Discussion section:

To determine how these different long-lived B cell subsets contribute to protection against *P. falciparum* infection, it would be important to analyze the connection between the cellular repertoire and plasma IgG. For *P. falciparum* antigens, a moderate correlation between memory B cell abundance and IgG titers has been observed for some merozoite antigens, but not for others (28, 44). This is in line with studies for other pathogens, that showed a correlation between the percentage of memory B cells and IgG titers for antigens from several viruses and bacteria (48-51), while other studies have reported the absence of such a correlation (51-54). The lack of a correlation between the magnitude of the memory B cell and the antibody response fits with the prevailing model that memory B cells and plasma cells are two independently controlled arms of the humoral immune system (55, 56). To determine the contribution of different memory B cell subsets to the recall response against *P. falciparum*, it would be interesting to analyze IgG responses upon re-infection. However, none of the individuals included in this study experienced a recorded *P. falciparum* infection post-IRS, preventing us from performing such an analysis.

b. We have added additional discussion about the types of antigens recognized by atypical B cells to the Discussion section:

Prior studies have shown that while atypical B cells harbor reactivity against *P. falciparum* antigens (9,18), they are also enriched for autoreactivity (43). Specifically, atypical B cells produce antibodies against the membrane lipid phosphatidylserine, which can induce the destruction of uninfected erythrocytes and contribute to anemia (44).

**Significance**

General assessment:

Strengths:
- Novelty in contrasting two different types of P. falciparum antigen responses at the B-cell level.
- The use of tetramers is a cutting-edge technique to assess this question.
- Analyses were thorough and found contrasting differences in antigen-specific B-cell populations (atypical vs classical) between these 2 antigens for the first time (to my knowledge).
- Well-written manuscript with clear data, methodology, and conclusions

Limitations:
- Missing serum/plasma antibody data to support their claim about long-lived humoral responses and reconciliation of ag-specific B-cell levels and ag-specific antibody levels in experiments and discussion.
- Limited N of 10 patients of the same gender (female), some population analyses had even fewer samples.
- Missing baseline levels for non-endemic uninfected control for B-cell populations for comparison.

- We have included a discussion about the correlation between plasma antibody and memory B cell responses in the Discussion section.

- We have clarified that some data points overlap in Figure 2, giving the impression that data from fewer than 10 individuals were shown.

- We have included baseline levels of 1) tetramer reactivity (Figure S1), 2) the size of B cell populations (Figure 2), and 3) expression of select markers (Figure 4).

Advance:
The study consolidates antigen-specific responses with the discovery of recently characterized populations (ex. atypical) and finds novel differences between two types of malaria antigen responses at the B-cell level and between specific populations responding differentially to these antigens. The findings are incremental (role of B-cell population in malaria-specific responses), conceptual (contrasting two types of B-cell antigen responses in the same infection), and clinical (finding significant differences in patients).

Audience:
This study will attract basic B-cell immunology scientists, infectious disease clinicians/scientists, vaccinologists, and both basic malaria immunology and clinical audiences.

Reviewer expertise:
Malaria, immunology, antibodies.

**Reviewer #3**

**Evidence, reproducibility and clarity:**
The authors analysed the antigen specificity and phenotypes of B cells during high P falciparum transmission and after a period of successful malaria control with IRS in Uganda. The gap between the two sampling time points is close to two years.

They use antigen probes for MSP1/AMA1 and CIDRalpha1, two antigens expressed at different stages of P. falciparum life cycle-merozoites and infected red cells, respectively. While MSP1/AMA1 are involved in the parasite's invasion of red blood cells, CIDRalpha1 is a domain of PFEMP1, a large family of antigenically variant proteins that mediates the sequestration of infected red cells in small blood vessels.

They found that the percentage of activated antigen-specific memory B cells declined with malaria control. However, detectable frequencies of antigen-specific memory B cells were retained after malaria control, which confirms earlier reports.

However, they also demonstrate that the phenotypic characteristics of memory B cells are associated with antigen specificity. The retained MSP1/AMA1-specific B cells were mostly CD95+CD11c+ memory B cells and FcRL5-Tbet- atypical B cells. In contrast, the retained CIDRalpha1-specific B cells were enriched among a subpopulation of atypical B cells.

These findings suggest differences exist in how the MSA1/AMA1 and CIDRalpha1 y are recognised and processed by the human immune system and how the immune response responds to them upon re-infection with P falciparum.


**Major issues affecting the conclusion:**
The findings and conclusions of this study, whilst positively exciting and informative, are based on the analyses of very few cells (at times). Even the authors themselves acknowledge this. I expect the authors to address this issue by toning down their reporting and conclusions (where appropriate). Ultimately, we need to have the confidence that these results are reproducible.

We appreciate the reviewer’s concern about the numbers of antigen-specific cells included in our analyses, which is an inherent limitation of this approach. However, we would like to point out that most analyses included a substantial number of antigen-specific B cells:

Figure 3D: 158 to 2,038 cells per group

Figure 4: an average of 26 to 184 cells per donor

Figure 5B: 55 to 508 cells per group

Figure 5C: 10 to 334 cells per group*

* The group with 10 cells is an outlier here. All other groups contain at least 104 cells. Because this one condition had such a small number of cells, we specifically mentioned this number in the text.

The numbers of cells for analyses shown in Figures 3D and 5B were already included in the figures. All the other numbers were mentioned in Table S3. To further clarify the number of cells included in each analysis, we have added the number of cells to Figures 4 and 5C.

To tone down our reporting, we have rephrased some of our conclusions, and now present our section headers in past tense to make these statements reflect our observation instead of a definitive conclusion. For example:

Conclusion: “The loss of MSP1/AMA1-specific and CIDRα1-specific B cells in the circulation was similar, but the phenotype of long-lived MSP1/AMA1-specific and CIDRα1-specific B cells appeared to differ.”

Section header: “Long-lived MSP1/AMA1-specific and CIDRα1-specific B cells differed in phenotype”

Finally, in the Discussion section, we have added a statement to our paragraph describing the limitations of our study to stress the importance of reproducing our findings:

All in all, it will be important to perform additional studies of the phenotype and functionality of long-lived B cells with specificity for *P. falciparum* antigens to reproduce and extend our findings.

**Minor comments:**
Figure 2D-I found this figure, and its presentation is unclear. Notably, using contour plots doesn't allow the reader to appreciate the density of the cells being presented.

To facilitate the interpretation of this figure, we have changed the plot type to a contour plot with density color gradient, and added the number of cells shown in each plot. (Please note that this panel has been renumbered to C.)

Figure 4 - label the y-axis.

The y-axis was labeled with “%”, which we have expanded to “% of B cells expressing marker of interest”.

**Significance:**
The study design-as outlined-allowed for the analyses of the specificity and phenotypic characteristics of residual P falciparum-specific memory B cells after 1.7 years of little to no P falciparum exposure. The cell phenotyping methods presented are also appropriate. However, antigen-specific cells are rare in blood circulation, and as the authors themselves acknowledge in the discussion, some of the results are based on very few cells. This means we cannot be sure all the results presented are reproducible.

Previous studies demonstrated that P falciparum memory B cells are maintained long after cessation of antigen exposure. However, few (if any) detailed antigen-specific and phenotypic analyses of the characteristics of P falciparum-specific memory B cells following a long period of no exposure exist. Thus, this study presents an incremental advance in our knowledge. In addition, the association of antigen specificity with cell phenotypes is a new concept in malaria immunology.
The research presented will greatly interest infectious disease immunologists and vaccinologists.

I am an infectious disease immunologist with substantial experience in malaria immunology.
