# Supplementary material for: Differences in phenotype between long-lived memory B cells against Plasmodium falciparum merozoite antigens and variant surface antigens
Source: PLoS Pathog. 2024 Oct 28;20(10):e1012661. doi: 10.1371/journal.ppat.1012661 (PMC11542837; doi:10.1371/journal.ppat.1012661)
Supplement: Attachment — Submitted filename: Reviewer Comments.docx [file ppat.1012661.s010.docx]

We thank the reviewers for their evaluation of our revised manuscript and constructive suggestions for improvement. Please find below our point-by-point response.

Reviewer's Responses to Questions

**Part I - Summary**
Please use this section to discuss strengths/weaknesses of study, novelty/significance, general execution and scholarship.

Reviewer #1: The manuscript by Reyes RA, et al., provides a phenotypic description of Plasmodium falciparum-specific (MSP1/AMA1 and CIDRAa1) B cells in Ugandan adults during a period of high transmission and a subsequent period of low transmission, with about 1.7 years between the two time points. This study design allowed for a numeric and phenotypic assessment of long-lived antigen-specific B cells. The authors have provided a well-written assessment of their observations, with most conclusions supported by the data. These data provide further insight into the presence and potential contribution of atypical B cells towards anti-Plasmodium immunity, the maintenance and phenotype of Plasmodium-specific B cells in the absence of continuous antigen exposure, and the impact of Plasmodium antigen distribution between merozoite and surface of infected red blood cells on the phenotype of antigen-specific B cells. Collectively, these data will be a valuable resource to the malaria immunology community.

Reviewer #2: As our tools expand, our ability to identify heterogenous populations of cell types increases. In the case of malaria, there is a high interest in determining if atypical MBCs generated as early as the first exposure to the parasite in humans can play a protective role in recall responses against the parasite. Here, the authors wanted to address how circulating memory B cell (MBC) populations against two different classes of antigens (merozoite proteins involved in invasion and variant surface antigen) resemble or differ from one another after a period of high Plasmodium transmission and after the absence of infection for a median of ~1.7 yrs. The novelty here involves the utilization of B cell tetramers specific for MSP1/AMA-1 and the CIDRa1 domain of PFEMP-1. Spectral flow cytometry was used for phenotyping the MBC populations in the blood of ten Ugandan patients who were involved in a P. falciparum transmission reduction program. Importantly, the authors found that the MBCs generated against the different antigens differed after IRS, with the CIDRa1-specific MBCs primarily resembling atypical MBCs. In contrast, the phenotype of the MSP-1/AMA-1–specific MBCs resembled that of cells primed for activation or atypical MBCs. Overall, the authors conclude that different parasite-derived antigens drive the production of different phenotypes of B cells. This is a finding that will be of interest to others in the field.
While this study provides new insight into the ability of different classes of parasite antigens to give rise to MBCs of varying phenotypes, the study was constrained by several limitations, including being able to address whether these different MBC phenotypes result in functional differences upon recall remained unanswered. Also, the authors did not compare the degree of somatic hypermutations accumulated in the BCR of the different antigen-specific MBC populations, as differences may exist. However, the ability to address the latter question could be due to the limited availability of cells from the patients. Nevertheless, the absence of these data detracts from the findings' overall significance, which is largely a phenotypic characterization of MBC populations, as acknowledged by the authors.

Reviewer #3: This manuscript by Evelien Bunnick and colleagues utilizes a longitudinal study to examine the longevity of memory B cells to both merozoite (MSP1 / AMA1) and the CIDRα1 domain of PfEMP-1 in the absence of continued transmission. The strengths of the work are the thorough analysis of the B cell population in human samples using tetramers and comprehensive flow cytometry, not an easy task given that it is difficult to detect antigen-reactive B cell subsets as they are generally much smaller populations, and the discovery of different subsets of memory B cells for merozoite epitopes vs the infected RBC membrane epitope.

The major weakness of the work in my opinion is that there are only 10 patient samples tested raising a potential issue regarding the replicability of the data. Having said that I would be supportive of publishing the data in this study as it adds something to the literature, in my opinion more than previous reviewers have stated.

**Part II – Major Issues: Key Experiments Required for Acceptance**
Please use this section to detail the key new experiments or modifications of existing experiments that should be absolutely required to validate study conclusions.

Generally, there should be no more than 3 such required experiments or major modifications for a "Major Revision" recommendation. If more than 3 experiments are necessary to validate the study conclusions, then you are encouraged to recommend "Reject".

Reviewer #1: Lines 276-288 – It is not clear the data support the broad conclusion that CD95 and CD11c are differentially expressed between MSP1/AMA1- and CIDRa1-specific long-lived memory B cells as the percentage of these antigen-specific B cells are not different at the pre-IRS time point. That said, these markers are different between these antigen-specific B cells at the post-IRS timepoint. If the conclusion of this data set is that post-IRS represents “long-lived memory B cells” then the claim is supported. If so, then the authors should make it clear that they are referring specifically to the expression of CD95 and CD11c on the post-IRS time point, which they believe represents long-lived memory B cells.

We indeed considered antigen-experienced B cells detected post-IRS to be long-lived. This is mentioned several times in the manuscript:

Introduction (lines 50-51): “…long-lived anti-parasite immunity (here defined as persisting for at least one year in the absence of exposure).”

Introduction (lines 96-99): “Analyzing antigen-specific B cells from these two time points allowed us to evaluate which *P. falciparum*-specific B cell subsets remained present in the absence of new infections and could thus be considered long-lived.”

Results (lines 183-184): “…but that long-lived *P. falciparum*-specific B cells are detectable in the circulation after more than a year without parasite exposure”

We have further clarified that the conclusions for the analysis of CD95 and CD11c expression in long-lived memory B cells are based on our observations at the post-IRS time point:

“Thus, CD95 and CD11c, but not other markers of durable immunity, seem to be differentially expressed between MSP1/AMA1-specific and CIDRα1-specific long-lived memory B cells present post-IRS.”

Reviewer #2: (No Response)

Reviewer #3: There are a couple of data presentation issues I think should be addressed that would help the reader. I would consider the most important omission is the number of cells. All conclusions are based on % of total B cells. However, to be prudent I would like to see the absolute number calculated from the number of circulating PBMCs / B cells. If the incidence of malaria transmission indeed has been reduced in the area are the number of PBMCs in each matched patient sample different at the 2 time points? The answer to this question may well be that it is not but I think it would be important to include this information in some way, perhaps in the supplemental information. Accounting for differences in numbers relative to % is important for interpretation purposes.

We have included the number of cells for each B cell population in Supplementary Table 3. The average total number of B cells evaluated per individual decreased from 52k pre-IRS to 41k post-IRS. This could reflect the decrease in immune activation post-IRS. However, it should be noted that this number is also influenced by other, technical, variables, such as the number of PBMCs aliquoted into each vial and cell viability.

Similarly I would like to see a slightly different representation of the % data to help with visual interpretation. The axis in Figure 2 are all different. Whilst the authors have likely chosen to represent the data in this way to make differences between the two sampling time points clearer, it is harder for the reader to compare the changes visually between the different B cell populations as they may be represented in the bloodstream.

We have changed the y-axes in Figure 2 from linear to logarithmic and use the same range for each panel. This allows a direct comparison between the size of different B cell populations, while still showing the differences between the two sampling time points.

**Part III – Minor Issues: Editorial and Data Presentation Modifications**
Please use this section for editorial suggestions as well as relatively minor modifications of existing data that would enhance clarity.

Reviewer #1: (No Response)

Reviewer #2: Several concerns were raised during the initial review of the manuscript; in the resubmission, the authors largely addressed these concerns by adjusting figures and tables, tempering their conclusions, and adding to their discussion.

Reviewer #3: in line 63 I would also mention IgM – IgM has an underappreciated role in protection and there are papers (Pepper /Crompton / Pierce amongst others) on IgM that should be briefly discussed (I understand why isotypes are not a major feature of the analysis in this study but nonetheless humoral immunity to malaria is not all about IgG). IgM memory cells also exist.

At the first instance of IgG in the Introduction section, we now also mention IgM. In addition, we have added references to Hopp et al., 2021, JEM, Thouvenel et al., 2021, JEM, and Boyle et al., 2019, Science Advances (refs. 3-5), which provide evidence for a protective role of IgM against malaria.

“People living in malaria-endemic regions are repeatedly infected by *Plasmodium* parasites and, as a result of these repetitive exposures, develop immunoglobulin (Ig) M and IgG responses against asexual blood-stage parasites that protect against disease [2–6].”

Other instances of “IgG” have been changed to the more general term “antibody” to include IgM, unless the statement referred to a publication that specifically reported on IgG responses.

Line 63: IgG responses => antibody responses

Line 75: IgG^+^ B cells => antigen-experienced B cells

Line 475: plasma IgG => plasma antibody responses

Line 480: IgG responses => antibody responses

In line 68 – PfEMP-1 is certainly the most studied but I am not entirely sure it is necessarily the most important. For clarity maybe it might be wise to mention it is not the only variant family in falciparum.

We have clarified that there are several families of variant surface antigens and have replaced the qualification “most important” with “most studied”.

“The second class of antigens comprises several families of variant surface antigens that are expressed by *P. falciparum* on the surface of the infected erythrocyte. The most studied of these are members of the *P. falciparum* erythrocyte membrane protein 1 (PfEMP1) family that mediate binding to endothelial receptors on the host microvasculature.”
